# Computer Vision Syndrome Among the General Population in the Eastern Region of Libya: Prevalence and Risk Factors

**DOI:** 10.7759/cureus.48777

**Published:** 2023-11-14

**Authors:** Hassan M Alturaiki, Jawad S Alnajjar, Ibrahim A Alibrahim, Fatimah A Almuhaysin, Menetallah W El Gaddafi, Mohammed A Almarzoq, Fatimah M Alturaiki, Shaikha S Aleid

**Affiliations:** 1 College of Medicine, King Faisal University, Alahsa, SAU; 2 College of Medicine, Libyan International Medical University, Benghazi, LBY; 3 Department of Emergency Medicine, King Faisal General Hospital, Alahsa, SAU; 4 Department of Ophthalmology, King Fahad University Hospital, Alkhobar, SAU

**Keywords:** risk factor, libya, computer use, digital eye strain, computer vision syndrome

## Abstract

Aim

To assess the prevalence and determine the risk factors of computer vision syndrome among the general population in the Eastern region of Libya.

Methods

This study used a descriptive cross-sectional design and comprised a random sample of 407 Libyan adults from Eastern Libya. Data were collected using an online-based questionnaire. Statistical analysis of all the datasets was performed using SPSS software, version 21.0 (IBM Corp., Armonk, NY).

Results

The mean age of the participants was 32.1 years (SD=12.8), and most participants 281 (69%) were female. As for employment status, 261 (64.1%) were students, 70 (17.2%) were non-healthcare workers, and 46 (11.3%) were healthcare workers. The majority of participants, 353 (86.7%), used a computer seven days a week, with 187 (45.9%) of those participants using a computer for over six hours daily. Over one-third of participants, 157 (38.6%), had computer vision syndrome. Being over 45 years of age, being a student, and using a computer for over six hours a day were the main factors associated with computer vision syndrome. Neck pain was the most commonly reported complaint in 235 (57.5%).

Conclusion

Increased use of electronic devices is leading to a higher prevalence of computer vision syndrome. Our study emphasizes the need to raise awareness regarding computer vision syndrome among the general public and medical professionals.

## Introduction

Electronic devices have become widely used in the 21st century and are an integral part of most people’s daily lives [1]. People spend more than 75% of their waking hours on computers [2]. People use many types of digital displays at home or the workplace. These include laptops, desktop computers, smartphones, and e-readers. If the time spent on these digital devices exceeds three hours per day or more than 30 hours per week, it increases their likelihood of developing computer vision syndrome (CVS) [3].

CVS, also known as digital eye strain or visual fatigue, is a set of ocular and visual disorders caused by prolonged exposure to high-resolution digital display terminals, such as computers, tablets, and smartphones, as defined by the American Optometric Association [4]. The reported number of CVS cases accounts for over 60 million individuals worldwide. In Ethiopia, Egypt, and Nigeria, the prevalence rate of CVS is 81.3%, 75%, and 54.2%, respectively [5]. In addition to the amount of time spent using digital devices, strobe lights, poor lighting, eye problems, advancing age, and improper sitting postures are also risk factors for CVS [6-11].

CVS is divided into four categories based on the symptoms. These are asthenopic CVS, ocular surface CVS, visual CVS, and extraocular CVS [7]. Although the discomfort associated with extensive computer use has not been proven to result in a long-term decline, it can reduce one’s skillfulness by almost 40%. Therefore, an increase in CVS prevalence is anticipated to result not only in increased health problems associated with CVS but also in a considerable decrease in workforce productivity [8].

Several studies have found a higher prevalence of CVS among certain professions. An association has been found between bankers, data processors, radiologists, secretaries, and CVS [9,10]. Numerous research studies have demonstrated the connection between dry eye syndrome (DED) and digital screen use (DSU). However, no such studies have been carried out in Libya [1]. Hence, this study aims to evaluate the prevalence and determine the risk factors for CVS in the general population of the Eastern region of Libya.

## Materials and methods

Study design and population

This descriptive cross-sectional design study was conducted on Libyan adults (>18) living in Eastern Libya and included the following cities: Benghazi, Al-Bayda, Darnah, Al-Marj, and Ajdabiyah. These cities were chosen because most of their sociodemographic and cultural characteristics are similar and their inclusion resulted in a broader study sample.

Research strategy and context

Using an online-based questionnaire, this study aims to explore the prevalence and risk factors of Computer Vision Syndrome (CVS) among the general population in Eastern Libya.

Sampling strategy

From March 2023 to April 2023, a random sample of Libyan adults was acquired from each of the above-mentioned Eastern Libyan cities. The questionnaire was sent online to the participants and was voluntarily filled out by all individuals who agreed to participate in the study. A total of 407 individuals had submitted the full questionnaire by the time data collection was complete.

Data collection material

The data was collected using a four-part structured questionnaire confirmed through consultation with experts. In the first portion, demographic information, such as nationality, gender, age, work status, and city of residence, was collected. Data on the respondents’ workstation characteristics were asked for in the second part. This included the type of electronic devices used, the type of lighting, the weekly usage of computer screens, and the daily duration of computer usage at work. The third part focused on the frequency and intensity of CVS-associated symptoms (CVS-Q). These 19 symptoms were rated by the subjects depending on their severity as mild, moderate, and severe/intense. The last part asked about carpal tunnel syndrome (CTS) diagnosis as well as important positive and negative symptoms. It also asked about the presence and effect of CTS symptoms during pregnancy (for female participants).

Statistical analysis

The data were collected, reviewed, and then fed to Statistical Package for Social Sciences (SPSS) software, version 21 (IBM Corp., Armonk, NY). All statistical methods used were two-tailed with an alpha level of 0.05. P-values ≤ 0.05 were considered statistically significant. Descriptive analysis was conducted by prescribing frequency distribution and percentages for study variables, including participants’ demographic data, computer use, associated CVS symptom frequency and intensity, and associated complaints. Ocular symptom frequency and associated complaints were graphed as well as the overall prevalence of CVS. Cross tabulation to assess factors associated with CVS among study participants was carried out with Pearson Chi-square test for significance and exact probability test if there were small frequency distributions.

## Results

A total of 407 participants fulfilling the inclusion criteria completed the study questionnaire. Participants’ ages ranged from 18 to over 45 years with a mean age of 32.1 ± 12.8 years. A total of 281 (69%) participants were females. As for employment, 261 (64.1%) were students, 70 (17.2%) were non-healthcare workers, and 46 (11.3%) were healthcare workers. As for residents, 187 (45.9%) were from Benghazi, while the others were from other cities (Table 1).

**Table 1:** Personal characteristics of study participants in the Eastern Region of Libya

Personal data	Frequency (n=417)	Percent (%)
Age in years		
< 25	175	43.0%
25–45	211	51.8%
> 45	21	5.2%
Gender		
Male	126	31.0%
Female	281	69.0%
Employment		
Unemployed	30	7.4%
Student	261	64.1%
Non-healthcare worker	70	17.2%
Healthcare worker	46	11.3%
Residence		
Benghazi	187	45.9%
Al-Bayda	66	16.2%
Marj	56	13.8%
Ajdabiyah	54	13.3%
Darnah	32	7.9%
Another city	12	2.9%

Nearly half of the study participants (49.1%) used smartphones, 89 (21.9%) used desktops, and 40 (9.8%) used tablets. As for lighting type, 202 (49.6%) used incandescent lighting, 200 (49.1%) used fluorescent lighting and 128 (31.4%) used natural sunlight. A total of 353 (86.7%) used a computer seven days per week, and among them, 187 participants (45.9%) did so for more than six hours a day and 150 participants (36.9%) for four to six hours a day. The pattern and frequency of computer use among study participants in the Eastern Region of Libya are shown in Table 2.

**Table 2:** Pattern and frequency of computer use among study participants, Eastern Region of Libya

Computer use	Frequency (n=417)	Percent (%)
Type of Computer used		
Smartphones	385	94.6%
Desktop	89	21.9%
Tablet	40	9.8%
Television	62	15.2%
Lighting Type		
Fluorescent	200	49.1%
Incandescent	202	49.6%
Natural sunlight	128	31.4%
Days of computer usage/week		
1–4 days	30	7.4%
5–6 days	24	5.9%
7 days	353	86.7%
Number of working hours/days		
1–3 hours	70	17.2%
4–6 hours	150	36.9%
> 6 hours	187	45.9%

Exactly 68.3% had eye burning, which was intense for 19.2% of the participants. Moreover, 63.6% had an itching sensation (22.2% intense), 53.6% had excessive tearing (19.3% intense), 52.3% experienced eye pain (17.9% intense), and 43.5% had eye redness (15.2% intense). The frequency and intensity of ocular symptoms among study participants are shown in Table 3.

**Table 3:** Frequency and intensity of ocular symptoms among study participants, Eastern Region of Libya Frequency: Never (the symptom does not occur at all); Occasionally (sporadic episodes or once a week); Always: Two or three times a week or almost every day. Intensity: Mild-Moderate-Severe.

Eye symptoms	Frequency n (%)			Intensity (%)		
	Never	Occasionally	Always	Moderate	Intense	
Eye burning	129 (31.7%)	215 (52.8%)	63 (15.5%)	329 (80.8%)	78 (19.2%)	
Itching	148 (36.4%)	205 (50.4%)	54 (13.3%)	317 (77.8%)	90 (22.2%)	
Foreign body sensation	251 (61.7%)	124 (30.5%)	32 (7.9%)	341 (83.7%)	66 (16.3%)	
Tearing	189 (46.4%)	168 (41.3%)	50 (12.3%)	328 (80.7%)	79 (19.3%)	
Excessive blinking	266 (65.4%)	112 (27.5%)	29 (7.1%)	306 (75.3%)	101 (24.7%)	
Eye redness	230 (56.5%)	145 (35.6%)	32 (7.9%)	345 (84.8%)	63 (15.2%)	
Eye pain	194 (47.7%)	179 (44.0%)	34 (8.4%)	334 (82.1%)	73 (17.9%)	
Heavy eyelids	255 (62.7%)	122 (30.0%)	30 (7.4%)	319 (78.5%)	88 (21.5%)	
Eye dryness	263 (64.6%)	106 (26.0%)	38 (9.3%)	301 (73.9%)	106 (26.1%)	

**Figure 1:**
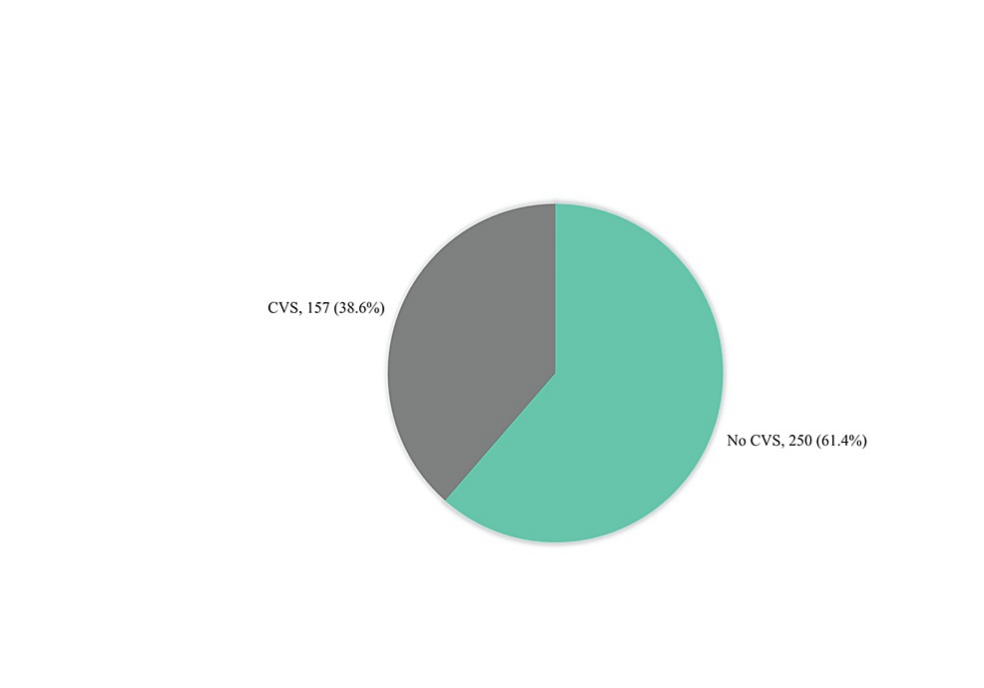
Prevalence of computer vision syndrome among study participants, Eastern Region of Libya

**Figure 2:**
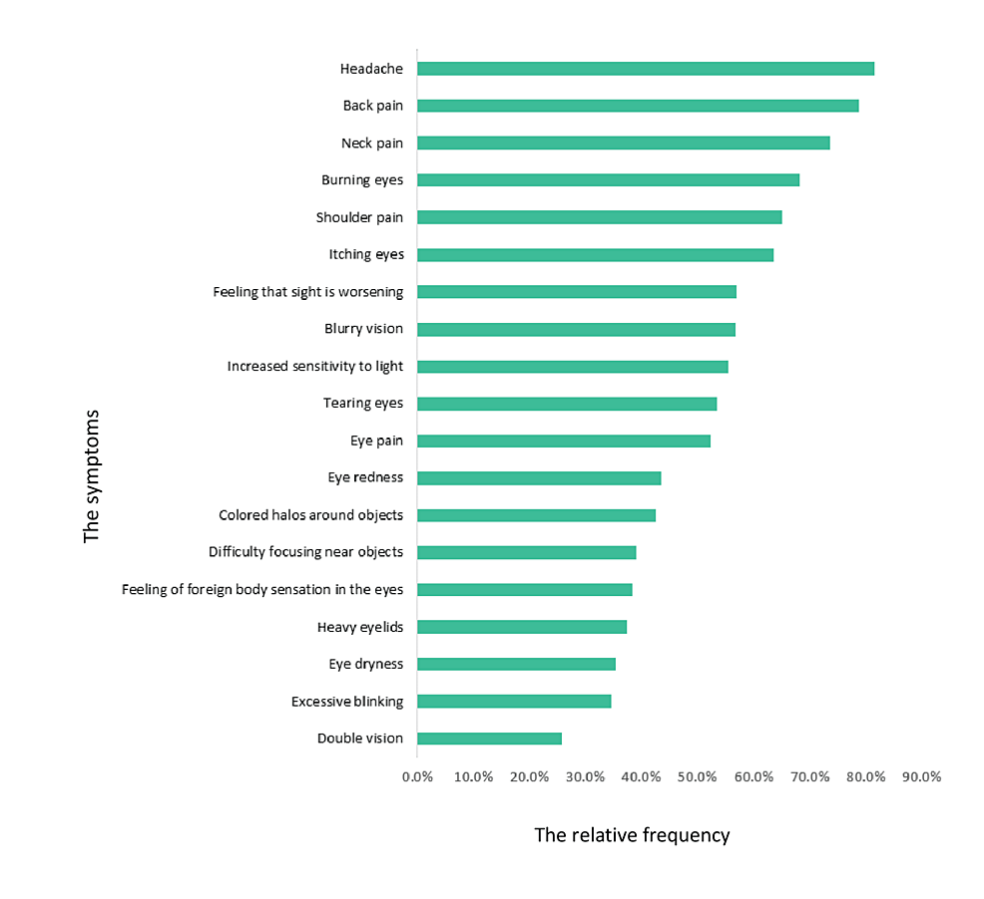
Relative frequency of symptoms associated with CVS among study participants, Eastern Region of Libya CVS: Computer vision syndrome

A total of 157 (38.6%) individuals were diagnosed with CVS, whereas 250 (61.4%) individuals exhibited no abnormalities. The most reported frequencies were headache (82%), back pain (79%), neck pain (74%), burning eyes (68%), shoulder pain (65%), itching eyes (64%), and increased sensitivity to light (56%). The prevalence of computer vision syndrome and the frequency of symptoms associated with CVS among study participants are shown in Figures 1-2.

A statistically significant difference was observed in the prevalence of CVS between the two age groups: specifically, 52.4% of participants over the age of 45 years had CVS, while only 28.4% of those aged 25-45 years were affected (p-value = 0.001). Furthermore, CVS was detected among 43.3% of students compared to 26.1% of healthcare workers (p =.049). Likewise, 46% of those who used computers for more than six hours daily had CVS compared to 30% of others who used computers for one to three hours daily (p =.016). The factors associated with CVS among study participants are shown in Table 4.

**Table 4:** Factors associated with computer vision syndrome among study participants P: Pearson X^2^ test; $: Exact probability test; * P < 0.05 (significant).

Factors	Computer Vision Syndrome				p-value
	No CVS		CVS		
	No.	%	No. %		
Age in years					.001*
< 25	89	50.9%	86	49.1%	
25-45	151	71.6%	60	28.4%	
> 45	10	47.6%	11	52.4%	
Gender					.566
Male	80	63.5%	46	36.5%	
Female	170	60.5%	111	39.5%	
Employment					.049*$
Unemployed	22	73.3%	8	26.7%	
Student	148	56.7%	113	43.3%	
Non-healthcare worker	46	65.7%	24	34.3%	
Healthcare worker	34	73.9%	12	26.1%	
Type of computer used					.347
Smartphones	240	62.3%	145	37.7%	
Desktop	50	56.2%	39	43.8%	
Tablet	23	57.5%	17	42.5%	
Television	36	58.1%	26	41.9%	
Lighting type					
Fluorescent	119	59.5%	81	40.5%	
Incandescent	120	59.4%	82	40.6%	.090
Natural sunlight	89	69.5%	39	30.5%	
Days of computer usage/week					
1-4 days	18	60.0%	12	40.0%	.933$
5-6 days	14	58.3%	10	41.7%	
7 days	218	61.8%	135	38.2%	
Number of working hours/days					
1-3 hours	49	70.0%	21	30.0%	
4-6 hours	100	66.7%	50	33.3%	.016*
> 6 hours	101	54.0%	86	46.0%	

The most frequently reported complaints were neck pain (57.5%), tingling and numbness when holding a mobile phone (50.4%), having/performing trick movements to make the tingling and numbness go from one’s hands (46.2%), and being woken by tingling and numbness in the hand during the night (35.1%). The associated complaints with CVS among study participants are shown in Table 5.

**Table 5:** Complaints associated with CVS among the study participants, Eastern Region of Libya

Items	Percent of "yes"
Has the pain in your wrist woken you at night?	66 (16.2%)
Has tingling and numbness in your hand woken you during the night?	143 (35.1%)
Are the tingling and numbness in your hand more pronounced first thing in the morning?	123 (30.2%)
Do you have/perform trick movements to make the tingling or numbness go from your hands?	188 (46.2%)
Do you have tingling and numbness in your little finger at any time?	95 (23.3%)
Have tingling and numbness occurred while you were reading a newspaper, steering a car, or knitting?	118 (29.0%)
Do you have any neck pain?	234 (57.5%)
Has the tingling and numbness in your hand been severe during pregnancy?	35 (8.5%)
Has wearing a splint on your wrist helped the tingling or numbness?	127 (31.2%)
Do tingling and numbness occur when you hold your mobile phone? 205 (50.4%)	

## Discussion

Our study aimed to assess the prevalence of CVS symptoms along with its risk factors among the Eastern Libyan population. The prevalence of CVS in our study group was 38.6%, which was 157 participants out of 407. The most frequently reported symptoms were non-ocular, with a high prevalence of headache (82%) and back pain (79%). This is in line with other previously published studies, which found headaches to be the most frequent symptom related to the use of computers [12-15]. In contrast, body fatigue was more common in computer use among bank workers in Pakistan, where half of the participants used the computer for more than eight hours a day, which could be explained by prolonged sitting during working hours [16,17]. The most commonly reported ocular symptoms in our study were eye burning and itching (68.3% and, 63.6%, respectively). The severity of eye itching severity was intense in 22.2% of the participants, and eye burning severity was intense in 19.2% of them. This finding is consistent with the above-mentioned study done in Pakistan, which found a 77.2% prevalence of eye burning [17]. Excessive blinking and eye dryness are the least reported symptoms in our study.

Our findings show a statistically significant association between being over 45 years of age and the development of CVS. A study in Sri Lanka showed similar findings, where the prevalence of CVS was higher among participants over 40 years of age (72.7%) [14]. This could be linked with decreased tear production as a normal process of aging [3]. Gender is not strongly related to CVS in our study. On the contrary, significant female predominance in the one-year prevalence of CVS was found in the study done in Sri Lanka [14]. Another study done in the United Arab Emirates among university students demonstrated some differences in gender with certain symptoms of CVS, and headaches were more frequently reported in females [8]. Moreover, a review done on CVS showed higher cases of dry eye in females [3]. A study on university administrative staff in Ghana reported a higher prevalence of CVS in males, but the author pointed out that this could be due to the unequal distribution of gender in their study sample [8].

In addition, there is a strong association between the prolonged use of computers for more than six hours a day and CVS in our study, which is reported to be 46% of CVS cases. This association was also observed in a study done in Nigeria, which showed that 48.9% of their participants with CVS symptoms were using the computer for six to eight hours a day [12]. Likewise, a study from Ethiopia reported a statistically significant association between prolonged daily exposure to the screen for more than 4.6 hours and the development of CVS [13]. Similarly, another study done among undergraduates in Nigeria and the study done in Ghana reported similar findings [15,18]. CVS symptoms in the present study were reported to be 41.7% of those who use the computer for five to six days a week, followed by 40% for using the computer one to four days a week. However, there is no significant association between the number of days of computer usage and CVS. The study done among undergraduates in Nigeria reported that the number of years of using the computer was significantly related to CVS and in that study, participants who used the computer for more than 7 years were more likely to get CVS [15].

The present study demonstrated a high prevalence of CVS in students. In accordance, a study done in India on medical and engineering students reported a high prevalence of CVS (80.3%) [19]. Another study done in Africa among undergraduates reported significant prevalence in students and clinicians [20]. This could be explained by the frequent use of smart devices nowadays for studying purposes. Lower cases of CVS are noticed in non-healthcare workers and unemployed participants in this study. On the other hand, the type of computer used, whether a smartphone, tablet, desktop, or television by the participants is not significantly related to CVS in the current study.

This study shows that the lighting type used in the room (i.e., fluorescent, incandescent, or natural sunlight) has no relation to the development of CVS. On the other hand, the study done in the United Arab Emirates among students reported that the intensity of lighting in the room was related to CVS symptoms; eye fatigue was higher when the room was extremely bright or extremely dark during the use of the computer [8]. Another related factor demonstrated by the study done in Sri Lanka was that the screen contrast, compared to the surroundings, was significantly related to CVS [14].

There were several limitations to our study. Firstly, the sample size was small. Secondly, we did not consider other factors such as the distance from which the text was viewed or the size of the font used. Thirdly, the symptoms reported were based on self-reporting, which could have resulted in biased data. Fourthly, we did not test whether wearing glasses had any correlation with CVS. Lastly, we did not examine whether the participants’ awareness played a role in the presence of CVS. To confirm our results, a large-scale, prospective, international study is required.

## Conclusions

The widespread disease known as computer vision syndrome (CVS) has started to become more noticeable worldwide. People are utilizing digital devices more frequently for work and entertainment, which has resulted in increased CVS prevalence. CVS can be accompanied by a variety of painful symptoms and can impair visual function. This study has explored the prevalence of CVS and its risk factors, such as excessive screen usage, bad lighting, and type of device. The results highlight the necessity of raising CVS awareness among the general public and medical professionals. The risk of getting CVS can be mitigated by employing techniques such as taking regular breaks and maintaining good posture. Further research is needed on treating CVS to identify the long-term health impacts of chronic digital device usage and determine the most optimal prophylactic strategies.

Overall, this study highlights the necessity of continuing research and teaching in this field and emphasizes the significance of identifying CVS as a crucial public health concern. We can minimize the burden of CVS and enhance people’s visual health and well-being in the digital age by cooperating to create efficient preventative and treatment techniques.

## Appendices

**Table 6:** Sociodemographic characteristics

Sociodemographic characteristics										
Nationality	Libyan ☐					Non-Libyan ☐				
Gender	Male ☐					Female ☐				
Age	<25 ☐			25-45 ☐				>45 ☐		
Work	Unemployed ☐	Students ☐	Teacher/Lecturer ☐		Healthcare worker ☐		Engineering ☐		Retired ☐	Others ☐
City	Benghazi ☐	Al-Bayda ☐	Almarj ☐		Ajdabiya ☐		Tobruk ☐		Darnah ☐	Other ☐

**Table 7:** Workstation characteristics

Workstation characteristics												
Type of Electronic Devices Used (You can choose more than one answer)	Computer ☐		Tablet ☐				Smartphone ☐				TV ☐	
Lightning Type (You can choose more than one answer)	Fluorescent ☐				Incandescent ☐				Natural sunlight ☐			
Days of computer usage/week	1 ☐	2 ☐		3 ☐		4 ☐		5 ☐		6 ☐		7 ☐
Number of working hours/day	1-3 h ☐				4-6 h ☐				>6 h ☐			

**Table 8:** Frequency and intensity of symptoms associated with CVS (CVS-Q) CVS: Computer vision syndrome Frequency: Never (the symptom does not occur at all); Occasionally (sporadic episodes or once a week); Always (two or three times a week or almost every day). Intensity: Mild/Moderate/Severe/Intense.

Frequency and intensity of symptoms associated with CVS (CVS-Q)			
Item		Frequency	Intensity
1	Back pain	Never ☐	Mild ☐
		Occasional ☐	Moderate ☐
		Always ☐	Intense ☐
2	Neck pain	Never ☐	Mild ☐
		Occasional ☐	Moderate ☐
		Always ☐	Intense ☐
3	Shoulder Pain	Never ☐	Mild ☐
		Occasional ☐	Moderate ☐
		Always ☐	Intense ☐
4	Burning eyes	Never ☐	Mild ☐
		Occasional ☐	Moderate ☐
		Always ☐	Intense ☐
5	Itching eyes	Never ☐	Mild ☐
		Occasional ☐	Moderate ☐
		Always ☐	Intense ☐
6	Feeling of foreign body sensation in the eyes	Never ☐	Mild ☐
		Occasional ☐	Moderate ☐
		Always ☐	Intense ☐
7	Tearing eyes	Never ☐	Mild ☐
		Occasional ☐	Moderate ☐
		Always ☐	Intense ☐
8	Excessive blinking	Never ☐	Mild ☐
		Occasional ☐	Moderate ☐
		Always ☐	Intense ☐
9	Eye redness	Never ☐	Mild ☐
		Occasional ☐	Moderate ☐
		Always ☐	Intense ☐
10	Eye pain	Never ☐	Mild ☐
		Occasional ☐	Moderate ☐
		Always ☐	Intense ☐
11	Heavy eyelids	Never ☐	Mild ☐
		Occasional ☐	Moderate ☐
		Always ☐	Intense ☐
12	Eye dryness	Never ☐	Mild ☐
		Occasional ☐	Moderate ☐
		Always ☐	Intense ☐
13	Blurred vision	Never ☐	Mild ☐
		Occasional ☐	Moderate ☐
		Always ☐	Intense ☐
14	Double vision	Never ☐	Mild ☐
		Occasional ☐	Moderate ☐
		Always ☐	Intense ☐
15	Difficulty focusing near objects	Never ☐	Mild ☐
		Occasional ☐	Moderate ☐
		Always ☐	Intense ☐
16	Increased sensitivity to light	Never ☐	Mild ☐
		Occasional ☐	Moderate ☐
		Always ☐	Intense ☐
17	Colored halos around objects	Never ☐	Mild ☐
		Occasional ☐	Moderate ☐
		Always ☐	Intense ☐
18	Feeling that sight is worsening	Never ☐	Mild ☐
		Occasional ☐	Moderate ☐
		Always ☐	Intense ☐
19	Headache	Never ☐	Mild ☐
		Occasional ☐	Moderate ☐
		Always ☐	Intense ☐

**Table 9:** The diagnosis of Carpal Tunnel Syndrome Cumulative percent of “yes” answers

Carpal Tunnel Syndrome (Diagnosis)			
1	Has the pain in the wrist woken you at night?	Yes ☐	No ☐
2	Has tingling and numbness in your hand woken you during the night?	Yes ☐	No ☐
3	Has the tingling and numbness in your hand been more pronounced first thing in the morning?	Yes ☐	No ☐
4	Do you have/perform and trick movements to make the tingling, numbness go from your hands?	Yes ☐	No ☐
5	Do you have tingling and numbness in your little finger at any time?	Yes ☐	No ☐
6	Has tingling and numbness presented when you were reading a newspaper, steering a car or knitting?	Yes ☐	No ☐
7	Do you have any neck pain?	Yes ☐	No ☐
8	Has the Tingling and numbness in your hand been severe during pregnancy? (female)	Yes ☐	No ☐
9	Has wearing a splint on your wrist helped the tingling and numbness?	Yes ☐	No ☐
10	Do tingling and numbness present when you hold your mobile phone?	Yes ☐	No ☐
